# A programme to spread eGFR graph surveillance for the early identification, support and treatment of people with progressive chronic kidney disease (ASSIST-CKD): protocol for the stepped wedge implementation and evaluation of an intervention to reduce late presentation for renal replacement therapy

**DOI:** 10.1186/s12882-017-0522-9

**Published:** 2017-04-11

**Authors:** Hugh Gallagher, Shona Methven, Anna Casula, Nicola Thomas, Charles R. V. Tomson, Fergus J. Caskey, Tracey Rose, Stephen J. Walters, David Kennedy, Anne Dawnay, Martin Cassidy, Richard Fluck, Hugh C. Rayner, Michael Nation

**Affiliations:** 1 South West Thames Renal Unit, Epsom and St Helier NHS Trust, Carshalton, UK; 2 UK Renal Registry, Bristol, UK; 3 University of Bristol, Bristol, UK; 4 School of Health and Social Care, London South Bank University, London, UK; 5 Renal Services Centre, Freeman Hospital, Newcastle upon Tyne Hospitals NHS Trust, Newcastle upon Tyne, UK; 6 PPI Representative for UK Kidney Research Consortium and National Institute for Healthcare Research, London, UK; 7 School of Health and Related Research, University of Sheffield, Sheffield, UK; 8 South of Tyne and Wear Clinical Pathology Services, Gateshead Health NHS Foundation Trust, Gateshead, UK; 9 Clinical Biochemistry, University College London Hospitals, London, UK; 10 Quality Improvement, East Midlands Clinical Networks & Senate, Leicester, UK; 11 Department of Renal Medicine, Royal Derby Hospital, Derby, UK; 12 Heart of England NHS Foundation Trust, Birmingham, UK; 13 Kidney Research UK, Peterborough, UK

**Keywords:** Chronic kidney disease, Renal replacement therapy, Quality improvement, Evaluation studies

## Abstract

**Background:**

Patients who start renal replacement therapy (RRT) for End-Stage Kidney Disease (ESKD) without having had timely access to specialist renal services have poor outcomes. At one NHS Trust in England, a community-wide CKD management system has led to a decline in the incident rate of RRT and the lowest percentage of patients presenting within 90 days of starting RRT in the UK. We describe the protocol for a quality improvement project to scale up and evaluate this innovation.

**Methods:**

The intervention is based upon an off-line database that integrates laboratory results from blood samples taken in all settings stored under different identifying labels relating to the same patient. Graphs of estimated glomerular filtration rate (eGFR) over time are generated for patients <65 years with an incoming eGFR <50 ml/min/1.73 m^2^ and patients >65 years with an incoming eGFR <40 ml/min/1.73 m^2^. Graphs where kidney function is deteriorating are flagged by a laboratory scientist and details sent to the primary care doctor (GP) with a prompt that further action may be needed.

We will evaluate the impact of implementing this intervention across a large population served by a number of UK renal centres using a mixed methods approach. We are following a stepped-wedge design. The order of implementation among participating centres will be randomly allocated. Implementation will proceed with unidirectional steps from control group to intervention group until all centres are generating graphs of eGFR over time.

The primary outcome for the quantitative evaluation is the proportion of patients referred to specialist renal services within 90 days of commencing RRT, using data collected routinely by the UK Renal Registry. The qualitative evaluation will investigate facilitators and barriers to adoption and spread of the intervention. It will include: semi-structured interviews with laboratory staff, renal centre staff and service commissioners; an online survey of GPs receiving the intervention; and focus groups of primary care staff.

**Discussion:**

Late presentation to nephrology for patients with ESKD is a source of potentially avoidable harm. This protocol describes a robust quantitative and qualitative evaluation of a quality improvement intervention to reduce late presentation and improve the outcomes for patients with ESKD.

## Background

**Fig. 1:**
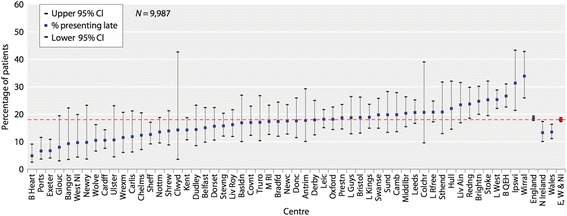
Variation in rate of late presentation to renal services across renal centres in the UK (2013–14). Reproduced with the kind permission of the UK Renal Registry [6]

Chronic kidney disease (CKD) is a common long-term condition that affects up to 14% of adults in England [1]. All patients with CKD are at increased risk of cardiovascular disease [2], and an important minority will develop progressive renal disease. In the most severe cases (end-stage kidney disease, ESKD), treatment with kidney dialysis or transplantation (renal replacement therapy, RRT) is required to sustain life. ESKD is strongly associated with increased mortality [3]. Although survival rates on RRT have improved over the last 15 years, the relative risk of death in RRT patients in the UK in 2013 across all ages was 6.2 compared with the general population [4]. Outcomes are particularly poor in those who present to renal centres with insufficient time to prepare adequately for RRT; the risk of death in such patients is approximately doubled as compared with those referred earlier [5], as a consequence of factors including greater use of temporary vascular access and delayed referral for transplantation. Late presentation, which is a term conventionally applied in the case of patients first seen by renal services within 90 days of starting RRT, is therefore an important cause of avoidable harm. In 2013–2014, the proportion of patients presenting late varied across UK renal centres from 4.9 to 33.9%, see Fig. 1 [6]. Reducing this variation is an important target for quality improvement activities. Of note, acute kidney diseases can develop *de novo* and cause ESKD within 90 days, accounting for 28% of overall late presentation [6]. Therefore it is impossible to avoid late presentation entirely, but this should be minimal for the majority of patients with non-acute renal disease.

A system of graphical surveillance of kidney function over time (“eGFR graphs”) has been operating at the Heart of England Foundation Trust in people attending the diabetes service since 2004 and across the entire population served by the Trust since 2012. It is the central part of a community-wide CKD surveillance system that has been associated with a decline in the incident rate of renal replacement and the lowest percentage of patients referred late (most recently 4.9%) for dialysis in the UK [6, 7]. The eGFR graph system identifies patients who are at high risk of requiring renal replacement therapy and who otherwise may not have received timely specialist attention. It is perceived by general practitioners to be easy to use and helpful in improving patients’ management [8]. A similar service implemented in the Kaiser Permanente system in Hawaii has led to improved outcomes mirroring those reported from HEFT [9].

## Aims and objectives

The overarching aim of ASSIST-CKD is to drive large-scale, measurable and sustainable change that reduces the burden of chronic kidney disease across the UK, and in particular reduces late presentation for RRT. We propose to achieve this through the phased implementation, across locations, of routine surveillance and reporting of eGFR graphs by pathology laboratories, thereby facilitating the early recognition and timely referral in primary care of patients with deteriorating kidney function.

## Methods

### Project intervention

Graphical surveillance of all estimated glomerular filtration rate (eGFR) results analysed within a participating pathology laboratory, and the reporting in graphical form of results deteriorating over time to the requesting clinician.

**Fig. 2:**
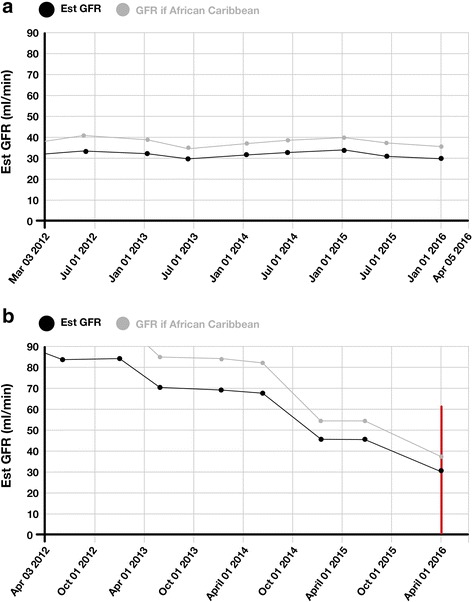
Examples of the graph intervention generated in the laboratory (**a**) low but stable function and (**b**) declining function (Graph 2b would generate an alert for the clinician as indicated by the red line)

A dedicated laboratory database operating on an SQL server integrates blood samples taken in all settings, i.e., community and hospital, and merges results stored under different identifying labels relating to the same patient. Graphs of eGFR over time are automatically generated for patients aged ≤65 years with eGFR <50 ml/min/1.73 m^2^ and patients >65 years with eGFR <40 ml/min/1.73 m^2^ using a dedicated software package, see Fig. 2 for examples. As eGFR results can be variable and unpredictable over time, the graphs are reviewed by a laboratory scientist or renal nurse. Graphs where the kidney function is clearly deteriorating are flagged.

The graphs from flagged patients are sent to the referring general practitioner, together with a prompt that further action, such as referral to specialist renal services, may be needed. These prompts can be adapted to meet local needs. The graphs are presented in a format that is meaningful to patients, and practitioners are encouraged to share the materials with patients to help increase their understanding of kidney disease. The laboratories are encouraged to include links to educational resources for primary care on progressive CKD with the graphs. A log of the patients on whom graphs are sent is kept.

### Education

Educational and training materials (both project-specific and generic) are available to participants. Learning events are being held at regular intervals throughout the life of the project, to bring together members of the project team and collaborators from the participating centres to share experiences and learning.

#### Laboratory staff

A pack for participating labs has been developed. This includes a role description for the laboratory staff reviewing the graphs, a Standard Operating Procedure, a User Guide, including a “YouTube” summary of how to run the software and interpret the graphs, and a summary of the IT specification. For external quality assessment, all individuals at participating sites who will be reviewing the graphs are invited to report an anonymised test set of 30 patients’ graphs supplied by the team at HEFT. The test set included graphs that are clearly progressive, stable and more nuanced. The outcome for each interpretation was agreed by consensus among consultant nephrologists and clinical scientists after independent review. Before live reporting can commence the reviewers are required to correctly flag all 10 of the subset of compulsory graphs showing clearly progressive CKD and in addition achieve a score of 80% overall.

The model used at the Heart of England Foundation Trust (HEFT) is for graphs to be reviewed by a Band 7 Clinical Scientist on the Health and Care Professions Council register. Review by a renal nurse may be possible in some locations. Oversight and supervision is provided by consultant Clinical (or Medical) Scientist. For sites where other models are proposed these will be examined on a case-by-case basis by the project team; in all cases a period of formal training and directly observed working followed by sign-off by the local consultant Clinical (or Medical) Scientist lead will be required.

#### Primary care staff

e-learning resources for primary care are freely available (http://www.ckdonline.org/ and http://www.theisn.org/education/education-topics/general-nephrology/item/2420-free-book-chapters-understanding-kidney-diseases).

#### Patients

An information booklet on CKD to inform patients and carers about what the results of an eGFR test might mean is freely available on-line and practices will be made aware of it (https://www.kidneyresearchuk.org/health-information/resources/looking-after-your-kidneys).

### Project design

It is planned that the eGFR graph intervention will be implemented in laboratories serving at least nineteen renal centres across the UK. All sites participating in this quality improvement programme will receive the intervention. However implementation will proceed in a phased stepped wedge manner (“stepped wedge cluster randomised trial”) to allow a robust outcome evaluation to be conducted. Without this design, evaluation of the overall project success may be overly optimistic due to the phenomenon of “early-adopters”.

The unit of intervention in the project is the pathology laboratory. However project outcome measures are aggregated at the level of the renal centre. Where more than one laboratory lies within the catchment of a main renal centre, where possible the intervention will be implemented in all these laboratories simultaneously.

The intervention will be rolled-out sequentially, with renal centre clusters (and their associated pathology laboratories) receiving the intervention at staggered time points. The plan is for four or five renal centres to begin implementation at each “step”, with a six-month interval between steps. The time of intervention initiation will be randomised. Crossover is unidirectional (from control to intervention) so eventually all participating centres will implement the intervention, see Table 1 for a representation of the step-wedge process.

**Table 1:** Study period with randomisation steps

Centres	Historical control-periods				Periods of core-study					
	(4 × 6 months)				Jan-Jun	Jul-Dec	Jan-Jun	Jul-Dec	Jan-Jun	Jul-Dec
	Jan-2013 - Dec 2014				2015	2015	2016	2016	2017	2017
A – D	0	0	0	0	0	1	1	1	1	1
E – I	0	0	0	0	0	0	1	1	1	1
J – N	0	0	0	0	0	0	0	1	1	1
O – R	0	0	0	0	0	0	0	0	1	1

0=’control’; 1=’intervention’ - eGFR graph surveilence

### Approach to adaptations during the project

This is a quality improvement project and adaptations according to local need are expected. We recognise that there may be delays in securing agreement from participating sites, and difficulties with Information Technology. There are a number of different Laboratory Information Management Systems (LIMS) in use in the UK, and only one of these (“Telepath”) has currently been interfaced with the project software.

We will have a total of four steps, each of six months duration. In summer 2015 we performed an initial randomisation (using random number generation) to an intervention start-time. Due to delays and uncertainties with candidate sites, we performed a first randomisation to select the four centres in the first intervention step (from ten available at that time). A second randomisation to allocate the remaining 15 centres to the second, third and fourth randomisation steps was then performed in late 2015.

### Recruitment of renal centres

We have preferentially targeted renal centres: with good completeness of late presentation rate in existing UK Renal Registry data returns; that are served by a single laboratory or LIMS; and that have a high proportion of patients currently presenting late for RRT (as reported by the UK Renal Registry).

A predefined process for recruitment was followed, starting with email contact with renal centres and laboratories and following up with telephone conversations. Inclusion in the randomisation to allocate an intervention start time was performed once we had a formal expression of interest from both renal clinicians and laboratory scientists together with an indication from sites’ Information Technology departments that the technical requirements could be met.

Participating renal centres agree to: submission of vascular access data to the annual UK Renal Registry Multisite Dialysis Access Audit; submission of UK Renal Registry data in a timely fashion; and to provide the UKRR with the total number of new out-patient renal referrals (in-centre and outreach clinics) per month. The UK Renal Registry has Section 251 approval from the Health Research Authority to collect and hold identifiable information without individual patient consent, for patients with CKD stages 2–5, for audit and quality assurance purposes. The UKRR have permissions to hold patient-identifiable data and will hold the final dataset in a partnership agreement with Kidney Research UK. Access to the data will not be limited, except patient or centre identifiable information.

Laboratories will be required to either keep a log of all patients (containing individual level identifiers) for whom an eGFR graph is sent to the GP.

### Support for participating sites

The project grant includes the initial support necessary for laboratories to link their pathology systems to the SQL database and install the software. Training will be provided for local laboratory staff and staff costs for one year will be covered. The Heart of England Foundation Trust experience suggests the annual costs are in the order of £12,000 per year for a large laboratory serving a population of one million people. It is expected that the on-costs from year two onwards will be met locally. From the outset we have built in mechanisms to achieve sustainability, working with participating sites to develop business cases for local commissioners. The business case focuses on the savings derived from avoidance of dialysis being re-allocated to pay for the ongoing provision of eGFR graph surveillance. Experiences from HEFT suggest that delaying dialysis for one patient for one year will fund the graph surveillance for a population of 300,000 people for approximately 5 years.

### Outcome measures

Outcomes will be examined at renal centre level and, if necessary, laboratory level using Geographic Information Systems to map patients to laboratories. Routinely collected baseline data will be provided by the UK Renal Registry and baseline referral rates supplied by participating centres.

The UK Renal Registry (UKRR) routinely collects and publishes treatment and clinical performance data for all adult renal centres in the UK [10]. As part of this, it routinely receives an extract of data on numbers of patients starting dialysis in each renal centre and timeliness of referral.

Our primary quantitative outcome measure is the incidence of late presentation for renal replacement therapy, defined as any patient first seen by renal services within 90 days of starting renal replacement therapy. These data will aggregate into six-month time periods, two per calendar year (Jan to June; July to December).

The following secondary outcomes will be studied:

(i.) the use of temporary vascular access for starting dialysis
(ii.) latest eGFR measurement, within two weeks before start of RRT
(iii.) mortality at 6 months from start of RRT in new RRT patients
(iv.) the incident rate of ESKD, measured annually.

All outcomes measures are routinely collected by the UK Renal Registry with the exception of vascular access, which is collected annually as part of the Registry’s Multisite Dialysis Access Audit.

The number of new patient referrals (per quarter) will be recorded as a balancing measure. These data are readily extractable from renal centre appointment systems.

### Statistical analysis plan

#### Number of participants

We will present numbers of patients flagged to GPs by each laboratory, per time-period. We will present the number of patients referred to renal centres and number of new RRT patients, and these will be presented by renal centre per time-period. We will also provide a flow chart of the number of incident RRT patients that will be included in the analysis after excluding those with missing data.

#### Descriptive statistics

The characteristics of individuals by exposure (control versus intervention) will be presented for each renal centre.

#### Analysis of primary outcome

Analysis of changes in risk of late-referral will be performed using a mixed-effects logistic regression, as a patient level analysis, with the clustering at renal centres being accounted for by fitting renal centre as a random effect. The primary outcome response will be binary (patient referral <90 days = 1, patient referral ≥90 days = 0). It is expected that the risk of being late referred should decrease with the intervention. The odds ratio estimate of the risk of being late-referred for the treatment effect (intervention versus control) with 95% confidence interval will be presented (model 1). Analysis will be adjusted by time-period or step (model 2), and individual patients’ characteristics such as age at start of RRT, gender, primary renal diagnosis, ethnicity and deprivation score (model 3). Due to the nature of the intervention it is anticipated that its impact on late referral will change over time, as the risk of being late-referred is expected to decrease after the intervention is implemented, and possibly more so with time from start of intervention (we expect a cumulative effect, at least up to a certain point). Therefore we aim to explore for possible interaction between time and treatment effect (model 4).

The results from model 2 will be considered the primary result, as our intention is to determine if changes in the risk of being late-referred for RRT are related to the intervention and not to an independent time-trend.

Power calculations for the primary outcome have been derived on Stata [11], based on UKRR data returns from twelve candidate renal centres. The UKRR reached complete coverage of the UK in 2007 and data on adult incident RRT patients have been routinely collected since then. Therefore we are able to use data on new incident patients commencing RRT before the project started, and we plan to include in the analysis the population of RRT incident patients in 2013–14 for the participating centres. We therefore used the formula published by Woertman, which can accommodate the inclusion of additional historic data [12]. The percentage of late referral in these twelve centres ranged from 9 to 35%, with an average of 18%. With power set at 80%, alpha at 0.05 and intra-class correlation (ICC) at 0.05 (based on analysis of UKRR 2013 data) we should be able to detect a reduction in late presentation rate from 18 to 11%.

#### Analysis of secondary outcomes

For the type of vascular access used at start of RRT, analysis will be conducted in the same way as the analysis of primary outcome (mixed-effect logistic regression of the binary outcome 0/1, where 0 = permanent access and 1 = temporary access). This analysis will be performed on all new patients starting RRT with any treatment modality and also restricting the cohort to patients starting RRT on haemodialysis.

Latest eGFR measurement preceding RRT start in new RRT patients will be analysed using a mixed-effects linear regression model. Transformation of the response variable ‘eGFR’ may be necessary to achieve a normal distribution prior to analysis. The same model building sequence will be used as for the logistic analysis of the primary outcome.

The analysis of the six months mortality in new RRT patients will be conducted in the same way as the analysis of the primary outcome, using a mixed-effect logistic model (binary outcome, 0 = alive after 6 months from start of RRT, 1 = died in the 6 months following start of RRT).

The incidence rate of RRT (expressed as number of patients starting RRT per million population), will be analysed using Poisson regression. This will be a cluster-level analysis, with one measure per time-period per renal centre.

### Qualitative evaluation

The aim of the qualitative evaluation is to understand the experiences of those directly involved in the intervention, including laboratory staff, primary care staff, clinicians in renal centres and patients. In addition, the experiences of commissioners in facilitating and monitoring the intervention will be sought. The qualitative evaluation plan is based on previous learning from feedback at the Heart of England Foundation Trust (HEFT) in October 2014.

#### Laboratory staff

One-to-one semi-structured interviews lasting 20–30 min will be conducted with 1–2 laboratory staff in each Trust, either face-to-face or by telephone. Questions will explore the experiences of being involved with the eGFR project, and will include discussion of: what specifically has worked/not worked; how far the training has equipped staff to undertake this new role; and potential recommendations for other labs who will be developing the system in the future. Data will be analysed using simple thematic analysis.

#### Primary care staff

We will conduct an electronic survey, via SurveyMonkey, of a sample GPs who have been sent an eGFR graph. The overall aim of the survey is to determine if the intervention is effective and how far it might impact on a patient’s clinical care. Questions asking about timeliness, usefulness and ease of interpretation will be answered on a Likert scale. Open questions asking about possible impact on patient care and recommendations for future enhancements to the eGFR system will be answered in free text. Quantitative data will be analysed using simple descriptive statistics; free text will be analysed by simple thematic analysis.

One GP surgery in each stage of the intervention roll-out will be approached for an in-depth evaluation. This will involve a focus group with a variety of staff in the practice (GPs, nurse practitioners, practice nurses, practice managers) who may be involved with the intervention. Questions will be open and will commence with ‘What is your experience of using the eGFR system in this practice?’, but will move to further questions around timeliness, usefulness and patient experience. Focus groups will be recorded, data transcribed and analysed using simple thematic analysis.

#### Renal centre staff

Short face-to-face or telephone interviews will be requested and conducted with one nephrologist and one other staff member (eg. clinical nurse specialist or nurse consultant) who are involved in seeing patients who have been referred following GP review of the eGFR graph. Questions will focus on practicalities, such as barriers and enablers of adoption, and sustainability within the renal centre. Notes of the interviews will be taken and common themes identified.

#### Commissioners

Telephone interviews will be conducted with one CCG commissioner or quality improvement lead in each Clinical Commissioning Group (CCG)/Strategic Clinical Network (SCN). Interview questions will focus on impressions about barriers and enablers to adoption across the CCG/SCN, cost-effectiveness, sustainability and spread.

### Economic evaluation

The cost of implementing the intervention will be estimated from the study and existing published costs retrieved for renal outpatient attendances and renal replacement therapy options. Utility index values will be identified from the literature for the various health states possible and used to estimate cost per quality adjusted life year gained from the intervention. A NHS health care perspective will be adopted.

### Patient and public involvement

We have convened a Patient Project Team (PPT), following a successful model developed by members of the project team in a previous quality improvement project [13]. The PPT chair is an integral member of the Core Operational Team, providing patient leadership and contributing on an entirely equal footing to healthcare professionals. Terms of Reference and role descriptions were drawn up and signed off by team members. Members of the group will be reimbursed in accordance with National Institute for Health Research Guidelines [14]. The PPT will: guide and influence the development and delivery of all patient-facing materials within the project; work locally and nationally to facilitate adoption, spread and sustainability, using the power of the patient and carer voice to influence the commissioning process; work closely with healthcare professionals in the design and delivery of the project evaluation; and represent the patient and carer voice at national or local meetings.

### Project funding

Funding for the implementation of the ASSIST-CKD intervention for one year and a full quantitative and qualitative evaluation was awarded by the Health Foundation (Registered Charity 286967) to Kidney Research UK (Registered Charity 252892). Kidney Research UK will provide project governance and administration. The project is supported by the following partner organisations: Renal Association; British Renal Society, British Kidney Patient Association; National Kidney Federation and the Royal College of General Practitioners. The evaluation will be delivered through a collaboration between the School for Health and Related Research (University of Sheffield), the UK Renal Registry and London South Bank University.

The project has been funded by the Health Foundation as a part of their “Spreading Improvement” programme, the purpose of which is to support organisations to develop projects based on interventions developed with previous Health Foundation funding that have already demonstrated improvements and have potential to be transferable to realise greater benefits to the wider health service. However it is recognised that quality improvement projects may demonstrate initial success due to specific features of the local context and robust evaluation of a subsequent roll-out is then required to demonstrate continued efficacy outwith the context in which the intervention was originally developed. In addition, it is important to confirm that change does not merely reflect underlying temporal trends. The project was considered by the National Research Ethics Service (South East Coast-Surrey) and determined to be service evaluation, not requiring ethical review by an NHS Research Ethics Committee.

### Project oversight

An Advisory and Dissemination Board (ADB) has been established to provide directional stewardship to achieve spread and scale. The role of the ABD will be critical for dissemination and in linking the project to policy change. In addition, an Evaluation Advisory Group will oversee the project evaluation, ensuring that quantitative and qualitative elements are brought together to provide advice on risk/change management.

The project has been registered with the International Standard Randomised Controlled Trials Number (ISRCTN) Registry, registration number 13701669.

### Authorship

Those playing a major role in the design, conduct and evaluation of the intervention will be eligible for authorship of subsequent publications. These will be written by the research group and we will not use professional writers.

## Discussion

Guidelines on the identification and management of CKD that include referral criteria for primary care were issued by the National Institute for Health and Care Excellence in 2008 and revised in 2014 [15]. However cross-sectional data indicate that people with earlier but progressive disease are frequently not recognised and referred by primary care, and that many patients with more advanced but stable disease continue to attend hospital clinics where ongoing specialist input may result in little added value [16].

This suggests that there are significant opportunities for better interaction and integration between primary and secondary healthcare services in the UK to improve the quality and efficiency of kidney care. Late referral for renal replacement from primary care is an important cause of morbidity and mortality amenable to healthcare. Following the introduction of eGFR reporting and the inclusion of CKD within the UK pay-for-performance scheme for general practice there has been a steady decline in the proportion of patients presenting late for RRT, from 26–30% during 2000–2005 [17] to 18% in 2014 [6]. However the highest performing UK renal centres, which include the site at which the eGFR graph intervention was developed and tested, achieve late presentation rates of less than 10% [6]. Therefore evaluating the spread of the eGFR intervention to other centres is an appropriate focus for improvement activities.

Local evidence suggests that the graphical surveillance of kidney function is associated with measurable improvements in care [7, 8]. This project is applying a stepped wedge approach to the dissemination of the eGFR graph intervention, in order to scale up these improvements and to determine the effectiveness of the intervention away from the environment in which it was developed. The critical role of context in improvement work is well-described [18]. A stepped wedge design is particularly appropriate for the evaluation of interventions during routine implementation [19], and will complement the concurrent roll-out of the eGFR graph intervention across the West Midlands that is being funded by NHS England via the West Midlands Strategic Clinical Network and Clinical Senate [20].

Our statistical model balances the competing effects on power of the number of steps and the length of time between steps [21]. The quantitative analysis will be strengthened by the availability of a minimum of 2.5 years of high quality routinely collected baseline data at all sites, and supported by a qualitative element that will explore the barriers and enablers of change and the perceptions of the participants as described above.

The role of ethical oversight of quality improvement projects has been the subject of heated debate over recent years [22]. Recent reviews have emphasised a principle-based rather than a rule-based approach to determine whether an activity requires formal ethical approval, with the key elements including: the risks and benefits to existing or future patients; the need to respect individuals’ rights to self-determination; the preservation of privacy and confidentiality; and the distribution of the activity across patient groups [23]. The project team carefully considered these issues internally. It was recognised that the risks and burdens to patients were minimal. The project uses existing biochemical data within the laboratory and outcome measures are routinely collected. There is no allocation to different interventions across patient groups: due to the stepped-wedge design all participants receive the intervention by the end of the project. However, we also recognised that the project evaluation would generate new knowledge potentially relevant to the health service more widely. Accordingly we sought external advice, from both a former ethics chair and an independent expert. Their opinion was that the project appeared not to meet the criteria for research, but that a formal opinion from an Ethics Committee would be helpful. We therefore presented the project to the National Research Ethics Service for consideration (South East Coast-Surrey Committee): the Chair’s opinion was that the project was a service improvement/evaluation and did not require ethical approval.

Given the extreme financial constraints under which the UK National Health Service is currently operating, it is imperative to perform a robust evaluation of any quality improvement intervention. The proposed evaluation will ensure that the project is delivering improvement as expected and therefore represents an appropriate use of scant resource. It will also help to inform the aformentioned sites where the eGFR graph intervention is being introduced routinely outside the project [20].

The project has limitations and challenges. We recognise the complexities inherent in a stepped wedge approach, from both an analytical and operational perspective. The follow up is quite short and we do not have time to have a transition phase immediately following implementation of the intervention. We will not be performing interim analyses and believe that it is very unlikely that anything in these analyses would give good grounds to change the duration of the steps. However the number and duration of steps will be reviewed by a project Evaluation Advisory Board, if needed, as dictated by operational and logistical issues. There are several laboratory IT systems in operation across the UK and the need for the eGFR graph system to work across multiple platforms may impact upon the project time-lines. Delays will be mitigated by over-recruitment and pre-allocation of intervention start time at the point of formal expression of interest.

The UK pathology laboratory environment is evolving. The Audit Commission called for pathology services to be re-organised in 1993, but changes have been slow to be implemented. “Hub and spoke” models were recommended in Lord Carter’s 2008 review of pathology services, where routine (including GP) work is consolidated into large hubs. This model offers both opportunities and threats. Consolidation of high volumes of GP samples into a single laboratory/laboratory cluster will allow a single site deployment of our intervention to reach far larger numbers of surgeries. The risks are that historic pathology data may not be transferred to new systems, preventing the retrospective charting of renal function, and that there may be pathology reorganisations at participating sites within the project lifespan.

Routinely collected data are readily available and inexpensive, but have inherent weaknesses when compared with trial data: they can be incomplete and subject to bias, and may not collected or reported uniformly across different sites. Although the unit of intervention in the project is the pathology laboratory, UK Renal Registry data are aggregated and reported at the level of the renal centre. If sites are recruited where not all laboratories serving a main renal centre implement eGFR graph reporting, it is theoretically possible to map late presenting patients to laboratories using Geographic Information Systems; however this process is imperfect and subject to significant inaccuracies as a result of boundary effects.

We recognise that generating enthusiasm for improvement activity within the current financial envelope in the National Health Service may be challenging. Although participating sites will be fully supported to deliver the intervention for one year, achieving sustainability beyond one year will require support from local commissioners, which we anticipate will be contingent on evidence of the cost-effectiveness (or potential cost-effectiveness) of the intervention. Exploring the drivers and barriers to lasting change will be a key output of the qualitative element of our evaluation. It should also be recognised that there is an irreducible minimum late presentation rate as a result of renal failure that develops acutely, as seen for example with myeloma kidney [24].

The provision of an eGFR graph surveillance service is a conceptually simple quality improvement intervention that we believe should benefit both patients and clinicians. By its nature it is well-suited to replication at other sites. Set against the annual cost of haemodialysis, the system has clear potential to be highly cost-effective. A demonstration that it is self-sustaining and effective across a range of contexts would create a powerful case for universal adoption of the service.

## Abbreviations

ADB: Advisory and dissemination board
ASSIST-CKD: A programme to spread eGFR graph surveillance for the early identification, support and treatment of people with progressive chronic kidney disease
CKD: Chronic kidney disease
eGFR: Estimated glomerular filtration rate
ESKD: End-stage kidney disease
LIMS: Laboratory information management system
PPT: Patient project team
RRT: Renal replacement therapy
